# Circulating IGFBP-3 and Interleukin 6 as Predictors of Osteoporosis in Postmenopausal Women: A Cross-Sectional Study

**DOI:** 10.1155/2023/2613766

**Published:** 2023-03-31

**Authors:** Xiu Shi, Jingjing Jiang, Ru Hong, Feng Xu, Shouqian Dai

**Affiliations:** ^1^Department of Obstetrics and Gynecology, The First Affiliated Hospital, Soochow University, Suzhou 215006, China; ^2^Department of Obstetrics and Gynecology, The Affiliated Huai'an Hospital of Xuzhou Medical University and Second People's Hospital of Huaian, Huai'an 223001, China; ^3^Department of Emergency Medicine, The First Affiliated Hospital of Soochow University, Suzhou 215006, China; ^4^National Regional Center for Trauma Medicine, The First Affiliated Hospital of Soochow University, Suzhou 215006, China

## Abstract

**Objective:**

To explore the relationship between circulating IGFBP-3, IL-6, and bone mineral density and the potential diagnostic role of circulating IGFBP-3 and IL-6 in postmenopausal women with osteoporosis.

**Methods:**

Eighty-five postmenopausal women at Soochow University's First Affiliated Hospital, Osteoporosis and Menopause Clinics, were recruited. Forty-five of 85 women were diagnosed with osteoporosis. Circulating IL-6, PTH, 1,25(OH)2D3, osteocalcin (OST), IGF-1, IGFBP-3, and bone mineral density (BMD) of the lumbar spine (LS) and femoral neck (FN) were measured in 40 ordinary and 45 osteoporotic women. A simple regression analysis calculated the correlation between age, BMD, IL-6, and IGFBP-3. Multiple stepwise regression analyses were conducted to determine which variables were independently related to BMD. The potential role of IGFBP-3 and IL-6 in the diagnosis of postmenopausal osteoporosis was predicted using the area under the receiver operating characteristic curve (ROC, AUC).

**Results:**

Age, years since menopause, and circulating IL-6, PTH, and IGFBP-3 were significantly higher in the osteoporosis group compared to the normal group. Osteoporotic women had substantially lower BMDs of the LS and FN than normal women. Age-related increases were found for IGFBP-3 and IL-6, whereas age-related decreases were observed for LS/FN BMD. IGFBP-3 and IL-6 were both negatively correlated with LS and FN BMD. Stepwise multiple regression analysis showed that IGFBP-3 and IL-6 were strong predictors of BMD in postmenopausal women. AUC cut-off values (IGFBP-3: 3.65, IL-6: 0.205) were best evaluated for the diagnosis of postmenopausal women with osteoporosis, and the AUC for circulating IGFBP-3 and IL-6 were 0.706 (95% CI 0.594–0.818) and 0.685 (95% CI 0.571–0.798), respectively.

**Conclusion:**

In this cross-sectional study of postmenopausal women, IGFBP-3 and IL-6 were negatively related to BMD. Circulating IGFBP-3 and IL-6 might be essential predictors of postmenopausal osteoporosis and can help predict osteoporotic fracture.

## 1. Introduction

Postmenopausal osteoporosis is a relatively common metabolic disease in postmenopausal women, and the research regarding its pathogenesis is still sparse [1]. Studies have shown that estrogen deficiency is a fundamental cause of the disease [2]. The primary clinical basis for diagnosing postmenopausal osteoporosis is the bone mineral density (BMD) of the lumbar vertebrae and proximal femur. Postmenopausal women experience low back pain as their primary clinical symptom. Epidemiological surveys show that more than 10% of osteoporosis occurs in postmenopausal women, but fewer patients have fractures with the progression of the disease [3]. At the same time, the clinical manifestations of chronic pain in postmenopausal osteoporosis are not apparent, and there is a lack of sensitive indicators or predictors in early diagnosis [4].

However, in the past few years, some factors, particularly the IGF (insulin-like growth factor) system (IGF-I, IGF-II, and IGFBP- (IGF binding protein-) 1~6), have been proposed to play crucial roles in the pathogenesis of bone loss or osteoporosis in postmenopausal women. Especially, IGF-1 and IGFBP-3 play essential roles in regulating bone metabolism. IGF-I is a synthetic growth hormone secreted by the liver. Its prominent role includes stimulating osteocyte proliferation and inhibiting collagen degradation, thereby promoting bone growth and development [5]. IGFBP-3 is a binding protein of IGF-I, which regulates the synthesis of IGF-I, prolongs the half-life of IGF-I in bone metabolism, regulates the metabolism of vitamin D, and promotes the utilization of calcium [6]. According to a study, circulating levels of IGF-I and IGFBP-3 were significantly lower in older adults than in the young population, and a low circulating IGF-I and IGFBP-3 level was associated with osteoporosis [7]. However, other researchers reported that IGFBP-3 is a potent inhibitor of IGF-I-medicated DNA synthesis [8], indicating that IGFBP-3 could counteract the physiological function of IGF-I to a certain extent. In addition, Eguchi et al. demonstrated that IGFBP-3 might help maintain bone mass in both an IGF-I-dependent or IGF-I-independent manner by inhibiting osteoblast differentiation via the BMP-2 signal pathway [9]. Therefore, the specific role of IGFBP-3 in bone metabolism and osteoporosis remains controversial.

IL-6, a multifunctional cytokine, is secreted by activation of T cells, B cells, mononuclear macrophages, fibroblasts, specific tumor stromal cells, and osteoblasts. Although the content of IL-6 is minimal, it can act locally through autocrine and paracrine and affect the function of bone cells. It can promote the growth of hematopoietic stem cells, thereby exerting various biological activities [10]. A study involving 45 postmenopausal women showed that the circulating levels of IL-1*β*, IL-6, and TNF-*α* in postmenopausal women with osteoporosis were significantly higher than those without osteoporosis. At the same time, neither group had significant differences in other parameters of bone metabolism [11]. Scheidt et al. found that in women with osteoporosis, circulating IL-6 levels were positively correlated with bone loss in the first ten days after menopause, most notably in the hip [12]. This evidence suggests that postmenopausal osteoporosis is associated with IL-6.

This cross-sectional study is aimed at exploring the relationship between circulating IGFBP-3, IL-6, and bone mineral density in postmenopausal women. In addition, the potential diagnostic role of circulating IGFBP-3 and IL-6 in the pathogenesis of postmenopausal osteoporosis in postmenopausal women was investigated.

## 2. Materials and Methods

### 2.1. Population

Eighty-five postmenopausal women attending the bone mineral density examination at Soochow University's First Affiliated Hospital, Osteoporosis and Menopause Clinics, participated in this study. The circulating estradiol content in all patients was less than 20 pg/ml. Both healthy and osteoporosis women were included in our study. The age of menopause, duration of lactation, parity, and age of menarche were recorded for each participant. All participants have been out of menstruation for more than one year. We excluded patients with hepatic or renal dysfunction, thyroid disorders, or systemic diseases affecting bone metabolism. All participants gave informed written consent. The medical ethics committee of our hospital approved the study. All participants did not take medications known to affect bone metabolism.

### 2.2. BMD Measurements

The BMDs of the L2-L4 lumbar spine and femoral neck (g/cm^2^) were measured in all postmenopausal women using a dual-energy X-ray absorptiometry (DXA) system (Discovery, Hologic, Waltham, MA, USA). Among 85 postmenopausal women, 45 had osteoporosis (DXA T scores less than -2.5 standard deviations) according to the WHO criteria [13–15]. According to the in vivo variation coefficients, the lumbar spine had a variation coefficient of 1.7%, while the femoral neck had a variation coefficient of 2.2%. The same operator tested all the participants to eliminate operator discrepancies. In order to calculate the body mass index (BMI), body weight (kg) was divided by the square of body height (m^2^).

### 2.3. Biochemical Measurements

After an overnight fast, blood samples were collected, and serum was separated and stored at -20°C until testing. Standard automated techniques were used to perform routine serum determinations. The circulating IGF-I levels were measured using radioimmunoassay (RIA) after acid-ethanol extraction, and circulating IGFBP-3 levels were measured by RIA as described previously [16]. Circulating levels of parathyroid hormone (PTH), IL-6, osteocalcin (OST), and 1,25-dihydroxyvitamin D_3_ [1,25(OH)_2_D_3_] were measured as previously described [13, 17, 18]. These measurements were subjected to intra-assay variation of 2-3% and intra-assay variation of 6-7%, respectively.

### 2.4. Statistical Analysis

Means and standard deviations were calculated for all variables. This study used SPSS 26.0 (SPSS Inc., Chicago, IL, USA) for all statistical analyses. Comparisons were made between the normal and osteoporosis groups using an independent-sample *t*-test. Using Pearson's correlation coefficient, we evaluated the correlation between age, BMD, IL-6, and IGFBP-3 and determined the linear relationship using simple regression analysis. In order to determine which variables were independently related to BMD, multiple stepwise regression analyses were conducted. In order to determine whether IGFBP-3 and IL-6 circulating levels indicate osteoporosis in postmenopausal women, a receiver-operating characteristic (ROC) curve was constructed, and cut-off levels were selected. All analyses were considered statistically significant if the *p* value was less than 0.05.

## 3. Results

### 3.1. Baseline Characteristics

In this study, 85 postmenopausal women met the eligibility criteria for participation. The demographics and baseline data of the enrolled patients are presented in Table 1. Compared to the normal women, age, years duration of menopause, and circulating IL-6, PTH, and IGFBP-3 were significantly higher in the osteoporosis women. Compared to normal women, osteoporotic women had substantially lower BMDs at the lumbar spine and femoral neck. However, no significant differences in BMI and circulating levels of 1,25(OH)_2_D_3_, OST, and IGF-1 were observed in the two groups.

### 3.2. Age-Related Changes

**Figure 1:**
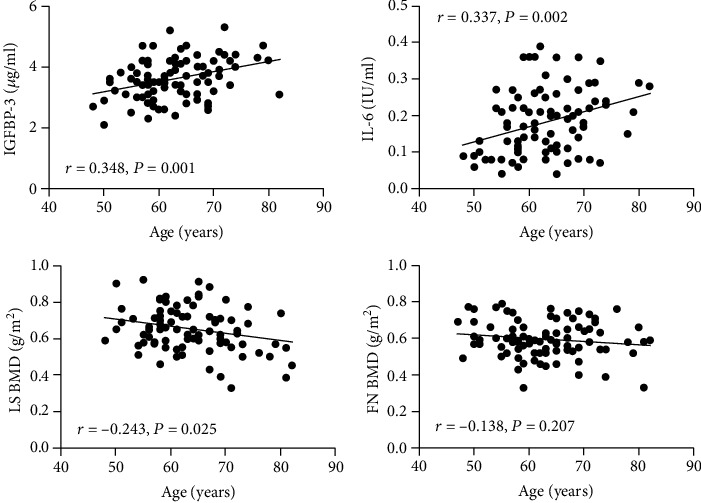
Correlations of age with IGFBP-3, IL-6, and BMD at the lumbar spine and femoral neck in all postmenopausal women. LS: lumbar spine; FN: femoral neck.

Correlations of age with IL-6, IGFBP-3, and LS/FN BMD in all study populations are presented in Figure 1. Age-related increases were found for IGFBP-3 (*r* = 0.348; *p* = 0.001) and IL-6 (*r* = 0.337; *p* = 0.002), whereas an age-related decrease was only observed for BMD of the lumbar spine (*r* = −0.243; *p* = 0.025). BMD of the femoral neck tended to decrease with age, but this correlation was not statistically significant (*r* = 0.138; *p* = 0.207).

### 3.3. Correlations with BMD

**Figure 2:**
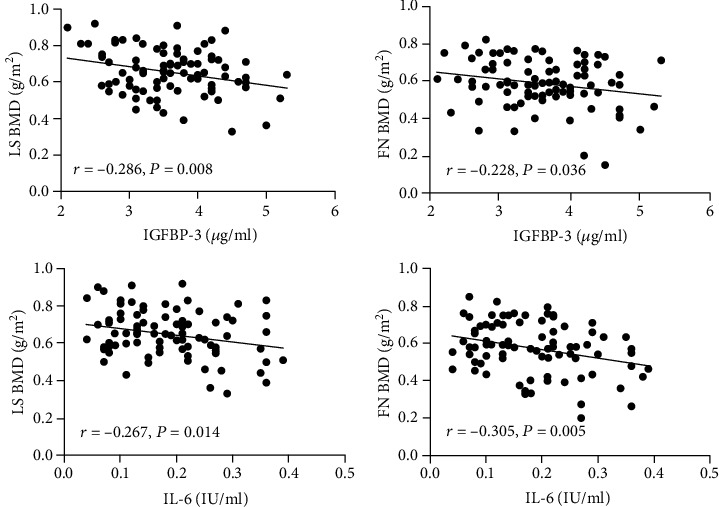
Correlations of LS BMD and FN BMD with IGFBP-3 and IL-6 in all postmenopausal women. LS: lumbar spine; FN: femoral neck.

Correlations of LS/FN BMD with IGFBP-3 and IL-6 in all postmenopausal women are presented in Figure 2. IGFBP-3 was negatively correlated with LS BMD (*r* = −0.286; *p* = 0.008) and FN BMD (*r* = 0.228; *p* = 0.036). Similarly, IL-6 was also negatively correlated with LS BMD (*r* = −0.267; *p* = 0.014) and FN BMD (*r* = −0.305; *p* = 0.005).

### 3.4. Determinants of BMD

A stepwise multiple regression analysis was conducted to identify the determinants of BMD by including age, BMI, IGFBP-3, and IL-6 as independent variables (Table 2). The multiple regression model included variables whose *p* values were 0.2 or less. Multiple regression analysis showed that age (*β* = −0.178; *p* = 0.015) and circulating IGFBP-3 (*β* = −0.304; *p* < 0.001) and IL-6 (*β* = −0.285; *p* = 0.004) levels are independent predictors of BMD of the LS BMD (*R*^2^ = 0.38). Meanwhile, age (*β* = −0.126; *p* = 0.011) and circulating IGFBP-3 (*β* = −0.328; *p* = 0.003) and IL-6 (*β* = −0.301; *p* = 0.012) levels were also independent predictors of BMD of the FN BMD (*R*^2^ = 0.32). IGFBP-3 and IL-6 were the strongest predictors of BMD in postmenopausal women.

### 3.5. Diagnostic Values of IGFBP-3 and IL-6

The ROC curve determined diagnostic values of circulating IGFBP-3 and IL-6 levels for postmenopausal osteoporosis. As shown in Table 3, the AUC for circulating IGFBP-3 and IL-6 was 0.706 (95% CI 0.594–0.818) and 0.685 (95% CI 0.571–0.798), respectively. When circulating IGFBP-3 and IL-6 levels of 3.65 *μ*g/ml and 0.205 IU/ml (Youden index 46.37 and 34.78) were taken as cut-off values, the sensitivity and specificity of their assay in the diagnosis of postmenopausal osteoporosis were 89.12% and 57.25% and 80.25% and 54.53%, respectively.

## 4. Discussion

The pathological features of osteoporosis include decreased bone formation, reduced bone mass and density, and destruction of bone microstructures. The main clinical symptoms are spinal deformity, diffuse bone pain, and fragility fractures. Currently, the risk factors of osteoporosis are relatively straightforward and multifactorial. Many studies have shown that smoking, lack of sunshine, a calcium-deficient diet, and some endocrine diseases increase the risk of osteoporosis in postmenopausal women [19]. However, there is no consensus on the relationship between circulating cytokines such as IGFBP-3 or IL-6 and new markers of bone turnover in postmenopausal women. This study compared the baseline characteristics of postmenopausal women in the normal and osteoporosis groups and found that the two groups had statistically significant differences in age; years since menopause; circulating IL-6, PTH, and IGFBP-3; LS BMD; and FN BMD. In our study, a significant correlation has also been found between circulating IGFBP-3, IL-6, and bone mineral density in postmenopausal women. Moreover, circulating IGFBP-3 and IL-6 levels were important potential diagnostic biomarkers for postmenopausal women with osteoporosis.

The estrogen hormone acts on the IL-6 promoter directly and on the IL-1 and TNF promoters indirectly and reduces the production of IL-1, IL-6, and TNF [20]. Postmenopausal osteoporosis is closely related to cellular senescence and inflammation caused by estrogen deficiency and is mainly characterized by enhanced osteoclast differentiation and bone resorption. Cellular inflammatory factors can regulate the formation of osteoclasts through the immune system and promote the occurrence and development of osteoporosis and pathological bone diseases. Previous studies have found that the knockout of the IL-6 gene could prevent bone loss after ovariectomy in mice. Applying IL-6 antagonists to transgenic mice with highly expressed IL-6 can prevent the occurrence of osteoporosis and growth retardation [21]. It has been reported that IL-6 can directly enhance osteoclast activity and inhibit its apoptosis, thereby prolonging osteoclast lifespan [22]. IL-6 can also promote osteoclastic activity and bone loss by activating the osteoprotegerin/receptor activator of nuclear factor kappa B ligand/receptor activator of the nuclear factor kappa B (OPG/RANKL/RANK) system, leading to osteoporosis [23]. In several animal models of chronic inflammation, estrogen can inhibit bone resorption and inflammation, reducing inflammation-mediated pain responses and the occurrence of neuroinflammatory diseases [24]. Our present findings are compatible with those of previous studies. There was a significant increase in IL-6 levels in the osteoporosis group compared to the control group in the current study. It was both negatively related to age and LS or FN BMD. Therefore, postmenopausal osteoporosis is significantly impacted by IL-6. Thus, exploring the relationship between IL-6, other inflammatory factors, and estrogen will deepen the understanding of the pathogenesis of osteoporosis.

As one of the most abundant IGFBP families (IGFBP-1~6) in blood circulation, IGFBP-3 is primarily produced in the liver and can transport more than 75% of IGF [25]. The physiological functions of IGFBP-3 include IGF transport and the regulation of the interaction between IGF and its receptors. IGFBP-3 can regulate the bioavailability of IGF by increasing its half-life and altering its biological activity on target tissues, thereby exerting physiological effects in an IGF-1-dependent or IGF-1-independent manner [26]. IGF-1 is a growth-promoting peptide substance induced by growth hormone, and it is also the most abundant growth factor in osteoblasts. Previous studies found that IGFBP-3 can antagonize the effect of IGF by combining with IGF to reduce the amount of free IGF. Therefore, it has antiproliferative, antimitotic, and proapoptotic physiological effects [27]. Our results indicated that the osteoporosis group had significantly high levels of IGFBP-3 than the control group.

Meanwhile, the circulating level of IGFBP-3 increased with age and was negatively related to both LS BMD and FN BMD. However, the results of some previous studies were inconsistent with our findings. Yang et al. found that IGFBP-3 could activate the growth factor signaling pathway and exert its inhibitory effect on osteoclasts [28]. Govoni et al. reported that the expression level of IGFBP-3 was significantly reduced with the aggravation of postmenopausal osteoporosis in female patients [29]. IGFBP-3 has both protective and destructive effects on the maintenance of bone mass. First, IGFBP-3 can act on target cells, including osteoblasts, by inducing apoptosis or programmed cell death [25]. Second, IGFBP-3 can inhibit the osteoblast differentiation induced by BMP-2 [9]. In addition, the overexpression of IGFBP-3 caused by increased RNA stability might be associated with aging-induced osteoporosis [8]. These findings suggest that IGFBP-3 promotes osteoporosis differently, but the specific mechanism needs to be explored.

According to the current stepwise multiple regression analysis, the independent predictors of LS and FN BMD were circulating IGFBP-3 and IL-6, which indicated that circulating cytokines were essential for preventing bone loss and osteoporosis. Other variables, including age, years since menopause, and PTH, did not predict BMD in postmenopausal women. However, they were significantly different in the normal and osteoporosis groups. An analysis of ROC curves showed that circulating levels of IGFBP-3 and IL-6 could be identified as reliable diagnostic biomarkers for postmenopausal osteoporosis.

There were several limitations in our study. First, our cross-sectional research was only observational at a single time, and our correlative data cannot be treated as definitive evidence of a causal relationship. Second, our study involved only a Chinese population at a single hospital, and there is a potential for selection bias in larger groups. Last, our sample size was not large, which might limit multiple variable analysis. Therefore, a prospective study or randomized controlled trial with a large sample size is recommended.

## 5. Conclusions

In this cross-sectional study of postmenopausal women, we found negative correlations between IGFBP-3, IL-6, and BMD in the lumbar spine and femoral neck. Circulating levels of IGFBP-3 and IL-6 explained between 28.5% and 32.8% of the variation of LS/FN BMD. Circulating measurements of IGFBP-3 and IL-6 might be essential predictors of postmenopausal osteoporosis and could predict osteoporotic fracture.

## Figures and Tables

**Table 1:** The demographics and baseline data of the participants in the normal group and osteoporosis group (mean and standard deviation).

Variables	Normal group (*n* = 40)	Osteoporosis group (*n* = 45)
Age (years)	60.9 ± 6.4	64.5 ± 7.6^a^
Years since menopause (years)	9.8 ± 2.1	12.4 ± 2.8^c^
BMI (kg/m^2^)	22.6 ± 3.3	22.1 ± 2.9
IL-6 (IU/ml)	0.15 ± 0.08	0.21 ± 0.09^b^
PTH (pg/ml)	29.4 ± 6.3	32.6 ± 7.1^a^
1,25(OH)_2_D_3_ (pg/ml)	26.3 ± 5.8	25.8 ± 4.7
OST (ng/ml)	7.3 ± 2.6	7.9 ± 3.1
IGF-1 (ng/ml)	178.5 ± 54.8	157.3 ± 45.9
IGFBP-3 (*μ*g/ml)	3.36 ± 0.56	3.87 ± 0.68^c^
LS BMD (g/cm^2^)	0.712 ± 0.108	0.636 ± 0.114^b^
FN BMD (g/cm^2^)	0.623 ± 0.086	0.568 ± 0.092^b^

Significant at ^a^*p* < 0.05, ^b^*p* < 0.01, and ^c^*p* < 0.001. BMI: body mass index; IL-6: interleukin 6; PTH: parathyroid hormone; OST: osteocalcin; LS: lumbar spine; FN: femoral neck.

**Table 2:** Stepwise multiple regression analysis between age, BMI, IGFBP-3, and IL-6 considering LS/FN BMD as dependent variables.

Variables	LS BMD			FN BMD		
	*β*	*P*	*R* ^2^	*β*	*P*	*R* ^2^
Age	-0.178	0.015	0.38	-0.126	0.011	0.32
IGFBP-3	-0.304	<0.001		-0.328	0.003	
IL-6	-0.285	0.004		-0.301	0.012	

BMI: body mass index; IL-6: interleukin 6; LS: lumbar spine; FN: femoral neck.

**Table 3:** Diagnostic value of IGFBP-3 and IL-6 for osteoporosis (*n* = 85).

Variables	AUC (95% CI)	Cut-off values	Sensitivity	Specificity	Youden index
IGFBP-3	0.706 (0.594-0.818)	3.65	89.12	57.25	46.37
IL-6	0.685 (0.571-0.798)	0.205	80.25	54.53	34.78

AUC (95%): area under the receiver operating characteristic curve (95% confidence interval).

## Data Availability

The datasets used during the current study are available from the corresponding author on reasonable request.
